# Dynamic Variation Patterns of Aconitum Alkaloids in Daughter Root of *Aconitum Carmichaelii *(Fuzi) in the Decoction Process Based on the Content Changes of Nine Aconitum Alkaloids by HPLC- MS- MS

**Published:** 2016

**Authors:** Heng Luo, Zhifang Huang, Xiaolong Tang, Jinhai Yi, Shuiying Chen, Andong Yang, Jun Yang

**Affiliations:** a *Centre of Instrumental Analysis, Sichuan Academy of Chinese Medicine Sciences, Chengdu, 610041, P. R. China. *; b *Institute of Traditional Chinese Medicine, Sichuan Academy of Chinese Medicine Sciences, Chengdu, 610041, P. R. China. *; c *College of Pharmaceuitcal Science,Chengdu University of Traditional Chinese Medicine, Chengdu, 611731, P. R. China. *; d *Funan Jinsha Community Health Service Center of Qingyang District, Chengdu, 610072, P. R. China.*

**Keywords:** Fuzi, aconite decoction, aconitum alkaloids, dynamic variation, HPLC- MS- MS

## Abstract

The chemical components in the decoctions of Chinese herbal medicines are not always the same as those in the crude herbs because of the insolubility or instability of some compounds. In this work, a high-performance liquid chromatography (HPLC) coupled with electrospray ionization (ESI) tandem mass spectrometry method was developed to explore dynamic variation patterns of aconitum alkaloids in Fuzi during the process of decocting aconite root. The fragmentation patterns of aconitum alkaloids using ESI and collision-induced dissociation (CID) techniques were reported. This assay method was validated with respect to linearity (r^2^ > 0.9950), precision, repeatability, and accuracy (recovery rate between 94.6 and 107.9%).The result showed that the amounts of aconitum alkaloids in the decoction at different boiling time varied significantly. In the decoction process，the diester- type alkaloids in crude aconite roots have transformed into Benzoylaconines or aconines.

## Introduction

Fuzi, the daughter root of *Aconitum carmichaelii *Debx. (Ranunculaceae), is official in Chinese Pharmacopeia (2010 version) (1). It has been clinically used for the treatment of rheumatism, neuralgia, and cardiac complaints for thousand years (2, 3). The following pharmacological effects of Aconitum alkaloid have been described: analgesic, anti-inflammatory, and anti-rheumatic activity; positive inotropic effects; and regulation of neurological disorders. However, the high toxicity risks and narrow therapeutic ranges limit the medicinal application on a larger scope. Typical symptoms of intoxication include rapid- onset facial and extremity paresthesias, chest discomfort, hypotension, and arrhythmias (4, 5). 

**Figure 1:**
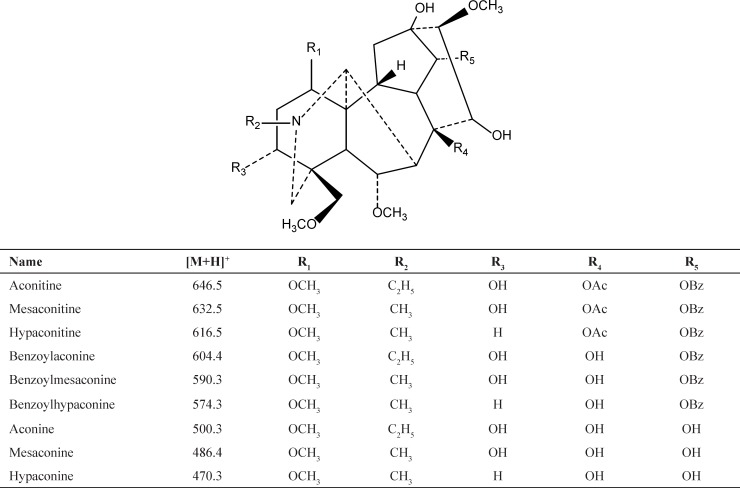
Chemical structures of 9 compounds

The main active components of Fuzi are aconitum alkaloids. Aconitum alkaloids consist of aconitines (diester-diterpenoid alkaloids), benzoylaconines (monoester- diterpenoid alkaloid) and aconines (amine alcohol-type alkaloids) as shown in Figure 1 (6). The high toxicity levels of aconitum alkaloids are considered to be derived from its aconitines. It is well-known that the processing of raw herbal materials is one of the characteristics of the Chinese medicine. Removing or reducing toxicity is the main objective for the processing of Fuzi. During processing, aconitines molecules lose their acetyl group at C_8_ and become benzoylaconines. The toxicity of the latter is 1/100–400 of the former. Benzoylaconines can further lose the benzoyl ester group at C_14_ to convert into aconines, whose toxicity is further reduced (6-8). Fortunately, this chemical reaction has been unconsciously applied for thousands of years in the Chinese medicine to reduce toxicity in the processing of aconite herbs. The processing of Fuzi is done by soaking, heating and decocting it in alkaline or water solution (4, 8-12). This process has no significant impacts on bioactivity and pharmacological effects (1, 13 and 14).

In fact, the toxicity of these herbs can be indicated by the amount of aconitum alkaloids. Therefore, the development of a rapid, valid, and sensitive method to simultaneously, qualitatively, and quantitatively assessing the aconitum alkaloids in Fuzi decoction is necessary and significant to ensure its safety and effectiveness in the areas of clinical drug use.

Many methods for the determination of aconitum alkaloids have been reported, such as high-performance liquid chromatography (HPLC) (15, 16), thin-layer chromatography (TLC) (17, 18), capillary electrophoresis (19). Nevertheless, these methods often required complete resolution of all constituents, which were time-consuming, and the sensitivities of these methods were very low. There have also been other methods, such as gas chromatography-tandem mass spectrometry (GC-MS) (20-23), liquid chromatography-fast atom bombardment (LC-FAB-MS) (24). However, the GC-MS (20-23) method used trimethyl-silyl derivatization and the extraction procedure was tedious. The LC-FAB-MS (24) methods achieved a high sensitivity, but the elution time was still very long. Liquid chromatography–mass spectrometry (LC-MS) (25, 26) and LC coupled with tandem mass spectrometry (LC-MS-MS) (27, 28) have been proposed for the determination of aconitum alkaloids in biological samples. However, all pharmacological and clinical studies mentioned above were related to raw aconite roots or biological samples without clear indications of Fuzi decoctions. It should be accepted that decocting is an easy way to reduce toxicity of Fuzi.

In the present study, we aimed to develop and validate a sensitive and accurate HPLC-MS-MS method for simultaneous determination of 9 aconitum alkaloids in the decoctions of Fuzi*,* including aconitine (AC), mesaconitine (MA), hypaconitine (HA), benzoylaconine (BAC), benzoylmesaconine (BMA), benzoylhypaconine (BHA), aconine, mesaconine and hypaconine. During the method development, multiple reaction monitoring (MRM) mode was employed and an electrospray ionization source was operated in positive mode. To the best of our knowledge, this is the most comprehensive report on the quantitative analysis of Fuzi decoctions. This paper explores dynamic variation patterns of aconitum alkaloids during the process of decocting aconite root so as to provide a reference for its further development and utilization.

## Experimental

*Chemical* *r* *eagents and* *m* *aterials*

AC, MA, HA, BAC, BMA, BHA, and Lannaconitine (IS) were purchased from National Institute for Food and Drug Control (Beijing, China). Aconine, Mesaconine, Hypaconine were extracted from Fuzi and purified in our laboratory. These compounds were identified using ESI-MS, ^1^H and ^13^C NMR techniques, and by comparing their experimental and reported spectroscopic data. The purity of all constituents is above 98% by HPLC analysis.

Six batches of Fuzi were purchased from Lotus Pond Chinese Medicinal Herbs Wholesale Market of Chengdu in Sichuan province, China, in November 2012, and authenticated by Prof. Guang-Ming Shu (the Department of Pharmacognosy, Sichuan Academy of Chinese Medicine Sciences (Chengdu, China)). HPLC-grade formic acid was purchased from Tianjin Kermel Chemical Reagents Development Center (Tianjin, China). HPLC-grade methanol was purchased from Fisher Scientific (Fair lawn, New Jersey, USA). Ultrapure water was prepared on a TCEH-RO/40 Reagent Water System (Beijing Aisitaike Technology Development Co., Ltd, Beijing, China) for the preparation of samples and buffer solutions. All other reagents were of analytical grade.

*Instrumentation and* *c* *onditions*

Chromatographic analysis was performed on an Agilent Rapid Resolution HPLC system, 1200 series (Agilent Corporation, MA, and USA) equipped with a binary pump, micro degasser, an auto plate- sampler, and thermostatic column compartment. Separation was performed on a Shiseido Capcell Pak C18 (3 × 100 mm, 3 μm) column. The mobile phase was composed of 0.1% formic acid (A) and acetonitrile (B), with a gradient elution as follows: 0 min: 12% (B), 3 min: 40% (B), 10min: 70% (B), 12 min: 80% (B), 12.01 min: 12% (B). The column temperature was set at 30 ˚C. The flow rate was 0.4 mL min^-1^.

Mass spectrometry was performed using an Agilent 6410 triple quadrupole mass spectrometer equipped with an electrospray ionization source (ESI) in the positive mode with the spray voltage set at 4,000 V. Nitrogen was used as nebulizer gas and nebulizer pressure was set at 40 psi with a source temperature of 105 ˚C. Desolvation gas (nitrogen) was heated to 350 ˚C and delivered at a flow rate of 10 L min^-1^. For collision- induced dissociation (CID), high purity nitrogen was used as collision gas at a pressure of about 0.15 MPa. Multiple reaction monitoring (MRM) mode was used for the quantification at *m/z* 646.5→586.4 for AC, *m/z* 632.5→572.5 for MA, *m/z* 616.5→556.5 for HA, *m/z* 604.4 →554.4 for BAC, *m/z *590.3→540.3 for BMA, *m/z *574.3→542.3 for BHA, *m/z *500.3→450.4 for Aconine, *m/z *486.4→436.2 for Mesaconine, *m/z *470.3→438.2 for Hypaconine, *m/z *585.4→356.3 for Lannaconitine (IS), Table 1 shows the optimized MRM parameters for detected drugs and IS. The peak widths of precursor and product ions were maintained at 0.7 amu at half-height in the MRM mode. Data acquisition was performed with Mass Hunter Workstation (Agilent Technologies, USA).

*Preparation of standard solutions*

Each accurately weighted standard was dissolved in methanol to give stock solutions. Working standard solutions containing 9 reference standards and IS were prepared by diluting the stock solutions with methanol- water (containing 0.05 M HCl) (4: 1, v/v).

*Preparation of* *its d* *ecoction* *sample*

Fuzi (100 g) were decocted in 1000 mL of water for 24 h (by boiling) by reflux extraction. The 1 mLof decoction was collected at 1, 15, 30, 60, 120, 240, 360, 480, 720 and 1440 min after boiling. The decoction was centrifugated at 12,000*g for 10 min and stored at 4 ˚C until use.

*Preparation of* *s* *amples for* *a* *nalysis*

A 0.5 mL of water extract (or standard solutions for calibration curve) and 0.5 mL of the IS working solution were mixed and diluted with methanol-water (containing 0.05 mol L^-1^ HCl) (4: 1, v/v) to 5 mL. Six batches of Fuzi decoction were subjected to HPLC-MS-MS analysis after being prepared. The solution was filtered by a 0.22 μm membrane filter. 5 μL of each filtrate was injected into the HPLC instrument for analysis.

*Method* *v* *alidation*

*Specificity*

The specificity was evaluated by comparing the chromatogram of blank (methanol- water (containing 0.05 mol L^-1^ HCl) (4: 1, v/v)) with the chromatogram spiked with respective standards to detect any peaks interfering the target compounds. 

*Linearity*

The linearity of the method was determined by plotting the peak- area ratios of the nine aconitum alkaloids to the IS vs. the nominal concentrations. The calibration curves were established by injecting each working solution twice. The linear regression with weighting factor of 1/x^2^ was applied as well as the slope (a), the intercept (b), and the correlation co-efficient (r) were determined from the regression analysis. The acceptable correlation coefficients were 0.995 or better. 

*Lower* *l* *imit of* *q* *uantification (LLOQ)* *and limit of determination (LOD)*

The LLOQ of the method was determined by spiking the lowest point of calibrator with precision and accuracy ≤ 20% which resulted in S/N ≥ 10: 1. The LOD was defined as the signal of the components can reliably distinguish from the background noise: S/N ≥ 3.

*Accuracy and* *p* *recision*

The accuracy and precision of the developed method were determined by the intra- and inter-day variations. For intra-day variability test, a sample solution prepared as the method described in Section *“Preparation of samples for analysis”* was analyzed for six replicates within one day, while for inter-day variability test, the sample was examined in duplicates for consecutive three days. The relative standard deviation (RSD) for peak area was calculated as the measure of precision and accuracy. The variation under 15% for the precision and accuracy was acceptable.

*Repeatability*

Five replicates of the same samples were extracted and analyzed. The RSDs were used to evaluate the method repeatability.

*Recovery*

Recovery was determined by analyzing spiked samples. A known amount of the standards (low, medium, and high concentrations) were added into a certain amount of samples (decoction of 30 min), and then prepared and analyzed with the same procedures. Three replicate extractives at each level were used to calculate the recovery rates for evaluating the method accuracy.

## Results and Discussion

*Optimization of LC conditions*

In order to achieve a rapid and high-throughput analysis of aconitum alkaloids, a fast HPLC coupled with a short column packed with 3 µm porous particles was employed. The chromatographic conditions were optimized systematically to improve the separation of the analytes. Different mobile phases (including methanol-water, acetonitrile-water, methanol-formic acid solution, and acetonitrile-formic acid solution) and flow rates (0.3, 0.4, 0.5 mL/min) as well as column temperatures (25, 30, 35, 40 ˚C) were examined and compared. As a result, acetonitrile- 0.1% formic acid solution at a flow rate of 0.4 mL/min with the column temperature of 40 ˚C resulted in satisfactory separation in a short analysis time.

*Optimization of MS* */* *MS* *c* *onditions*

Each investigated analyte was infused into the mass spectrometer, and the precursor ions and at least two product ions were preliminarily selected in both positive ion and negative ion modes. The results showed that all compounds exhibited excellent signal sensitivity in the positive mode. Then, the product ion and its fragmentary energy and collision energy were optimized to achieve the most abundant, specific, and stable transition for each compound. Finally, MRM scanning mode was established to quantify the target compounds in the samples. The optimum results are listed in Table 1. 

*Fragmentation* *p* *atterns of* *a* *conitum* *a* *lkaloids*

**Figure 2:**
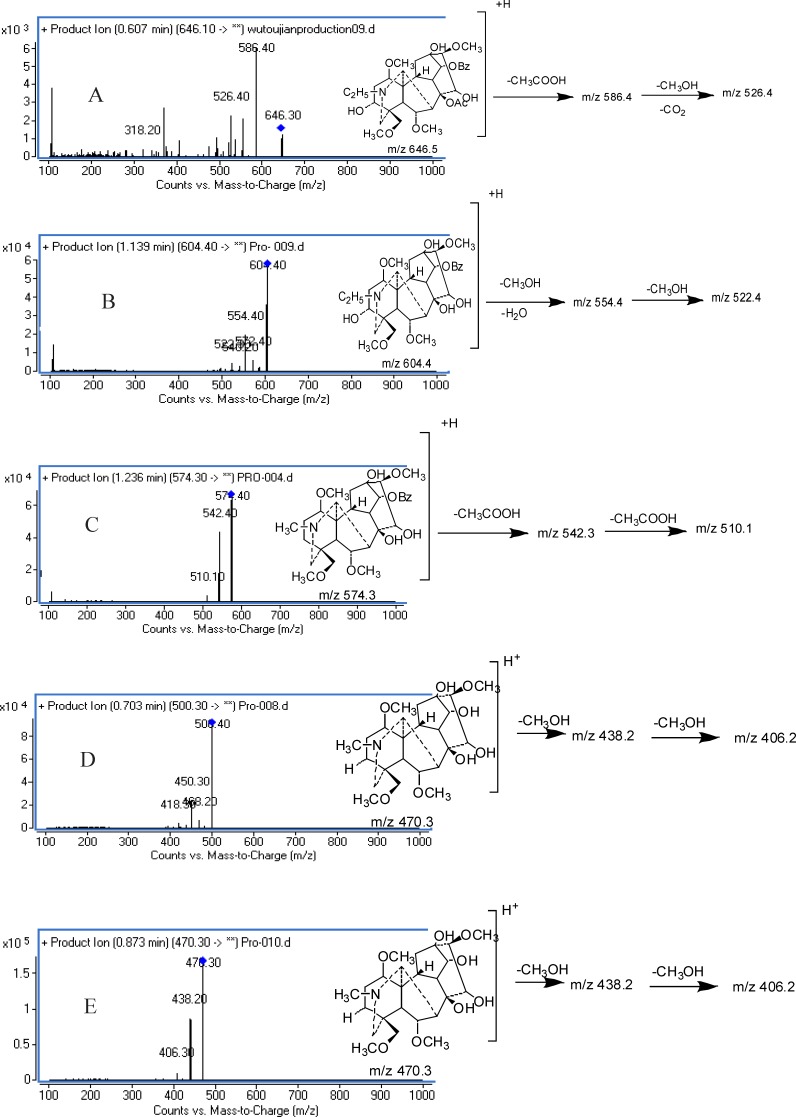
Product ion mass spectra of [M+H]^+^ and the proposed fragmentation pathways of AC (A), BAC (B), BHA (C), aconine (D), and Hypaconine (E

Fragmentation behaviors of aconitum alkaloids were analyzed. The product ion scans of five representative compounds are shown in Figure 2. It was found that the ESI- MS spectra were dominated by the presence of the [M + H]^+^ protonated molecule in the positive ion mode. Under CID conditions, for aconitines such as aconitine, the [M + H]^+^ ion at m/z 646.5 readily eliminated the acetyl (60 Da) moiety to produce the base ion at m/z 586.4. The second dominated product ion at m/z 554.3 was originated from the fission of a molecule of methanol (32 Da), and subsequently lost a –CH_2_CH_2 _moiety (28 Da) to give the ions of m/z 526.4. Similar fragmentation pathways were also observed in the MS/MS spectra of MA and HA (Figure 2A).

For benzoylaconines such as BAC, the [M + H]^+ ^at m/z 604.4 eliminated a molecule of methanol (32 Da) and water (18 Da) to produce the ion at m/z 554.4 , while the ion at m/z 522.4 was derived from the fission of a molecule of methanol (32 Da). BMA exhibited similar fragmentation behaviors to those of BAC (Figure 2B). 

The product ion scan of BHA displayed a base peak at m/z 542.3 derived from the loss of a molecule of methanol (32 Da) from the parent ion of m/z 574.4, The second abundant peak at m/z 510.1 was tentatively generated by the neutral loss of a molecule of methanol (32 Da) from the base peak ion (Figure 2C).

For aconine, the [M + H]^+ ^ion at m/z 500.4 readily eliminated a molecule of methanol (32 Da) and a water molecule (18 Da) to give the ions of m/z 450.4, and subsequently lost a molecule of methanol (32 Da) to give the ion of m/z 418.2. Similar fragmentation pathways were also observed in the MS/MS spectra of mesaconine (Figure 2D).

CID of Hypaconine yielded an ion at m/z 438.2 as the base peak, originating from the loss of a molecule of methanol (32 Da) from the parent ion of 470.4, subsequently lost a molecule of methanol (32 Da) to give the ions of m/z 406.3 (Figure 2E).

*V* *alidation of the method*

The calibration curves (the peak area versus the concentration of each analyte) were established by injecting each working solution twice. Limit of detection (LOD; S/N > 3), limit of quantification (LOQ; S/N > 10), precision, repeatability, and recovery were studied respectively as described below: The precision of the developed method was determined by the intra-and inter-day variations. For intra- day variability test, a sample solution prepared as the method described in “Preparation of samples for analysis” was analyzed for six replicates within one day, while for inter- day variability test, the sample was examined in duplicates for consecutive three days. The relative standard deviation (RSD) for peak area was calculated as the measure of precision. To confirm the repeatability, five replicates of the same samples were extracted and analyzed. The RSDs were used to evaluate the method repeatability. Recovery was determined by analyzing spiked samples. A known amount of the standards (low, medium, and high concentrations) were added into a certain amount of samples (1 min), and then extracted and analyzed with the same procedures. Three replicate extractives at each level were used to calculate the extraction recovery rates for evaluating the method accuracy.

The calibration curves exhibited good linearity (r^2^ > 0.9950) within the test range. The LODs and LOQs were less than 2.0 ng (Table 1). The intra- and inter-day variations (RSDs) of peak area for the 9 analytes were less than 3.2 and 5.7% (Table 2), respectively. The repeatability presented as RSDs were in the range from 1.78 to 4.56%. The recoveries varied between 94.6 and 107.4% with RSDs less than 4.42% (Table 3). The above data were considered to be satisfactory for subsequent analysis of all the samples. 

**Table 1:** Analytical parameters of the developed LC-MS-MS method

**Compound** **s**	**MS(m/z)**	**Precursor ion/product ion**	**Regression equation**	**R** ^2^	**Test range** **(ng/ml)**	**LO** **Q** **(ng/ml)**	**Fragmentor** **(V)**	**Collision energy** **(eV)**
AC	646.5	646.5→586.4	Y=71.437X+1.8624	0.9987	10.9-1089	0.28	210	40
MA	632.5	632.5→572.5	Y=75.265X-0.9023	0.9991	3.29-3294	0.31	150	38
HA	616.5	616.5→556.5	Y=82.962X+0.1404	0.9996	8.784-1728	5.1	170	39
BAC	604.4	604.4→554.4	Y=254.4X-5.0239	0.9999	52-2606	0.73	110	38
BMA	590.3	590.3→540.3	Y=365.08X-8.9833	0.9991	105-5252	0.29	120	35
BHA	574.3	574.3→542.3	Y=104.07X-9.5815	0.9985	572.9-11458	2.38	110	35
Aconine	500.3	500.3→450.4	Y=399.97X-3.0897	0.9997	13-3244	4.55	200	39
Mesaconine	486.4	486.4→436.2	Y=505.5X-2.1045	0.9997	10-4992	0.50	90	35
Hypaconine	470.3	470.3→438.2	Y=71.542X-8.1726	0.9989	69.1-6912	1.47	140	34

**Table 2:** The precision data of the proposed HPLC/MS/MS method

**Compound** **s**	**Nominal**	**Precision**			
	**Concentration** **(ng/ml)**	**Intra-day(n=** **6** **)**		**inter-day(n=3** **)**	
		**Mean ± SD** **(ng/ml)**	**RSD ** **(%)**	**Mean ± SD** **(ng/ml)**	**RSD ** **(%)**
AC	10.9 272 544.8	9.8±0.14 274.5±1.5 541±6.7	1.4 0.5 1.2	9.7±0.4 271.5±2.6 539.5±7.2	3.7 1.0 1.3
MA	13.2 329.5 1647	13.8±0.2 331±2.8 1645±9.8	1.1 0.8 0.6	13.4±0.4 334±3.7 1640±11.4	2.8 1.0 0.7
HA	8.832 88.32 702.7	8.99±0.2 92.92±2.9 700±3.9	2.1 3.1 0.6	8.74±0.3 91.02±3.7 685.2±4.9	3.2 4.0 0.7
BAC	130 651 2606	129.7±3.2 650.2±4.6 2609±10.6	2.5 0.7 0.4	127.5±4.7 654.8±6.7 2614±12.8	3.6 1.0 0.5
BMA	105 1313 5252	103.2±3.3 1311.5±8.9 5248±11.3	3.2 0.7 0.5	101.3±5.7 1308±12.7 5241±13.6	5.7 1.0 2.6
BHA	572.9 2864.6 5729.2	579.2±7.9 2860.3±9.3 5720.1±16.2	1.4 0.3 0.3	588±10.3 2857±11.3 5718.6±18.7	1.8 0.4 0.3
Aconine	32.4 324 1621.8	30.8±1.31 322.5±5.7 1618±7.2	4.2 1.8 0.4	31.2±2.5 319.2±6.8 1615±9.5	7.9 2.1 0.6
Mesaconine	39.9 691.2 3456	41.2±1.9 685.8±7.6 3450±11.1	4.6 1.1 0.3	39.8±2.3 688.3±6.2 3448±15.9	5.8 0.9 0.5
Hypaconine	138.2 691.2 3456	136.5±1.5 687.5±8.8 3442±14.1	1.1 1.3 0.4	134.9±1.9 684.1±9.5 3451±15.2	1.4 1.4 0.4

**Table 3:** Statistic results of recovery for extraction of analytes in aconite (n = 3).

Compounds	Original(ng/ml)	Spiked(ng/ml)	Detected(ng/ml)	Calculated recovery (%)
AC	30 30 30	11 22 44	43.2±1.1 55.6±2.3 72.8±1.4	105.4±2.8 106.7±4.4 105.7±2.0
MA	617 617 617	165 330 660	778.5±28.6 942.3±40.3 1265±33.0	99.6±3.7 99.5±4.3 99.1±2.6
HA	67 67 67	35 70 140	100.4±3.1 140.3±3.3 210.8±3.7	98.4±3.0 102.4±2.4 102.7±1.8
BAC	107 107 107	52 130 260	163.2±6.7 245.3±9.2 362±7.2	97.1±4.01 102.2±3.8 98.6±2.0
BMA	188 188 188	52.5 105 210	242±6.7 290±6.8 402.5±3.5	100.6±2.8 99.0±2.3 99.3±0.9
BHA	627 627 627	229 458 916	850±30.2 1095±33.7 1558±37.5	103.1±3.7 100.9±3.1 102.9±2.5
Aconine	25 25 25	13 26 52	41±1.7 53±1.9 74±2.2	107.9±4.6 103.9±3.7 96.1±2.9
Mesaconine	17 17 17	10 20 40	29±0.9 35±1.0 59±1.2	107.4±3.3 94.6±2.8 103.5±2.1
Hypaconine	64 64 64	28 56 112	90±3.7 124±4.1 168±3.7	97.8±4.0 103.3±3.4 95.5±2.1

*Quantification of 9* *c* *ompounds in the 6* *b* *atches of Fuzi*

*Raw* *m* *aterial and* *i* *ts* *d* *ecoction*

**Figure 3:**
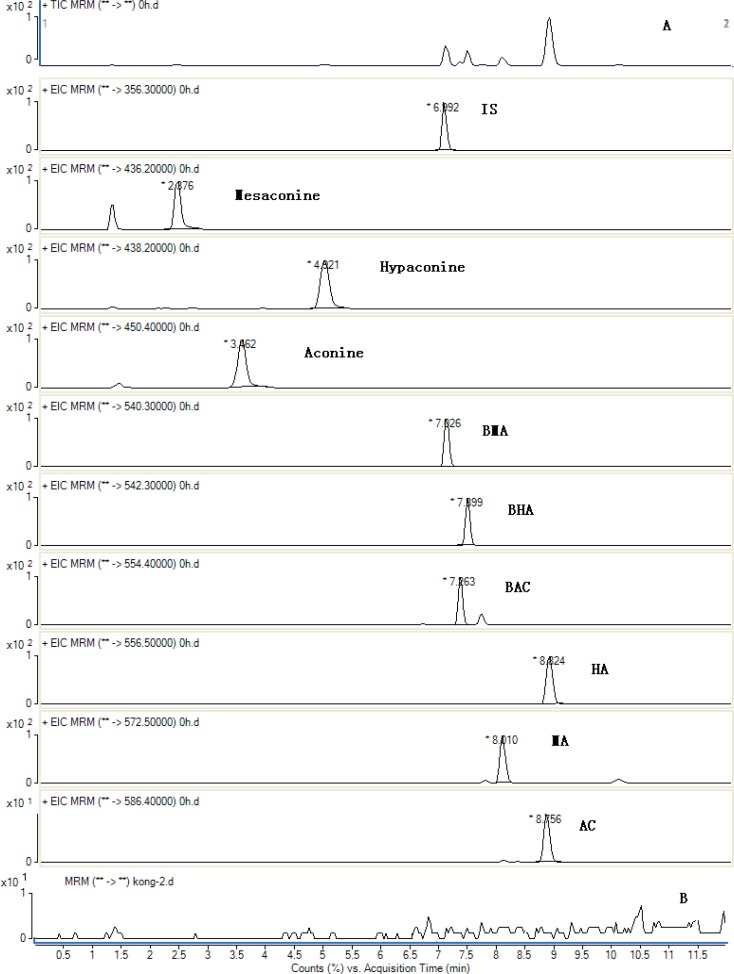
Total ion MRM chromatograms of the sample obtained in positive mode for IS and 9 compounds from the decoction at 1 min after boiling (A) and black (B).

**Figure 4:**
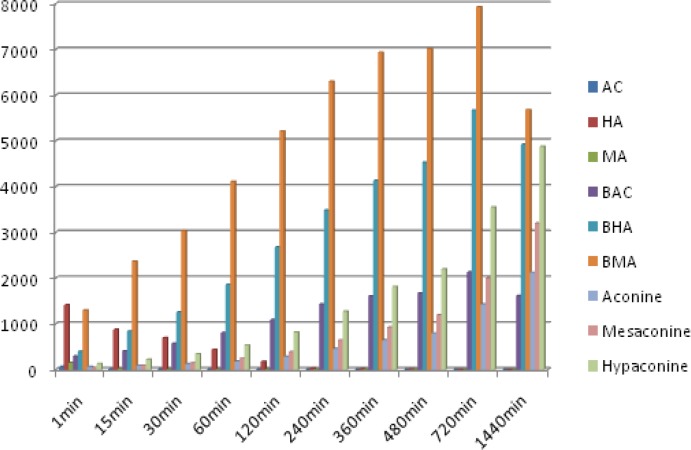
Amount of the 9 main components found in Fuzi decoction sample

The method was applied to quantitatively analyzes of 9 analytes in the 6 batches of Fuzi decoction at different boiling times. The typical MRM chromatograms are shown in Figure 3. The results of the contents of 9 analytes and the total contents of each type of compounds were calculated and the results are also listed in Figure 4. Comparing the amount of aconitum alkaloids in decoction, it was found that the amount of toxic components (aconitines) in decoction was significantly diminished. The contents of the highly toxic aconitines were significantly highest at 1 min, and could not be quantified at 1440 min. It was obvious that these constituents could be extremely unstable by decocting, while the relative amounts of the less toxic benzoylaconines increased markedly, reaching the peak level at 720 min, and then decreased at 1440 min. The contents of aconines were gradually increased within 1440 min. It indicated that the toxicity of Fuzi decoction was reduced drastically after decocting. This result indicates that decocting can improve the transformation of diester-diterpenoid alkaloids, increase the hydrolyzate content, and reduce drug toxicity. 

## Conclusion

In conclusion, FZ raw material contains a high amount of aconitines resulting in a strong toxicity. These toxic compounds can be decomposed during herb decocting. It is reasonable that herb decocting can reduce the toxicity and promote other therapeutic effects. This paper reveals dynamic variation patterns of aconitum alkaloids during the process of decocting aconite root so as to provide a reference for its further development and utilization. Moreover, the developed HPLC-MS-MS method for the quantitative analysis of the nine components in a large number of herb samples is rapid, accurate and reproducible; it is therefore practical and useful for batch-to-batch quality assurance of these toxic samples.
